# Expression of SARS-CoV-2 entry receptors in the respiratory tract of healthy individuals, smokers and asthmatics

**DOI:** 10.1186/s12931-020-01521-x

**Published:** 2020-09-29

**Authors:** Magdalena Matusiak, Christian M. Schürch

**Affiliations:** 1 Department of Pathology, Stanford University School of Medicine, Stanford, CA USA; 2 Department of Microbiology & Immunology, Stanford University School of Medicine, Stanford, CA USA

**Keywords:** SARS-CoV-2, COVID-19, ACE-2, TMPRSS2, Basigin, Furin, Smoking, Asthma, Respiratory epithelium, Coronavirus

## Abstract

SARS-CoV-2 is causing a pandemic with currently > 29 million confirmed cases and > 900,000 deaths worldwide. The locations and mechanisms of virus entry into the human respiratory tract are incompletely characterized. We analyzed publicly available RNA microarray datasets for SARS-CoV-2 entry receptors and cofactors *ACE2*, *TMPRSS2*, *BSG (CD147)* and *FURIN*. We found that *ACE2* and *TMPRSS2* are upregulated in the airways of smokers. In asthmatics, *ACE2* tended to be downregulated in nasal epithelium, and *TMPRSS2* was upregulated in the bronchi. Furthermore, respiratory epithelia were negative for ACE-2 and TMPRSS2 protein expression while positive for BSG and furin, suggesting a possible alternative entry route for SARS-CoV-2**.**

## Results & discussion

The current pandemic with the novel severe acute respiratory syndrome coronavirus 2 (SARS-CoV-2) that causes coronavirus disease 2019 (COVID-19) is spreading globally with more than 29 million cases and 900,000 deaths worldwide [1, 2]. In a significant fraction of patients, SARS-CoV-2 infection can take a severe course. Especially in the elderly and in those with pre-existing conditions including chronic lung diseases, severe pneumonia and even life-threatening diffuse alveolar damage requiring intensive care and ventilation can occur [3, 4].

The primary infection site for SARS-CoV-2 is the upper respiratory/digestive tract and conjunctival mucosa. The expression and distribution of SARS-CoV-2 entry receptors and cofactors in the human respiratory tract, and how their expression is altered in disease or by environmental and behavioral factors such as air pollution and smoking, is therefore of great interest. This will lead to a better understanding of SARS-CoV-2 biology, the susceptibility of certain populations to COVID-19, and potentially help to develop future therapies.

Angiotensin I converting enzyme 2 (ACE-2) and transmembrane serine protease 2 (TMPRSS2) have been described as the main receptor and cofactor for SARS-CoV-2 cellular entry [5–8]. In addition, emerging reports point towards a role for basigin (BSG / CD147) as receptor [9], and furin as a cofactor [10], in the pathogenicity and virulence of SARS-CoV-2. Here, we examined RNA and protein expression of ACE-2, TMPRSS2, basigin and furin in the human respiratory tract in healthy non-smokers, healthy smokers and asthma patients.

Six RNA microarray datasets of airway epithelial cell brushings, all generated with the Affymetrix Human Genome U133 Plus 2.0 Array, were downloaded from the Gene Expression Omnibus [11–16]. Affymetrix data files (Supplemental Information) were processed and normalized using the robust multiarray average expression measure method using affy and limma packages in R [17, 18]. For genes represented by multiple probes, the probe with the maximum average expression values in all samples was selected to represent that gene’s expression. First, by plotting the first 2 principal components computed on *ACE2*, *TMPRSS2*, *BSG* and *FURIN* expression across smokers’ and asthmatics’ datasets, we verified that there were no detectable batch effects within each of the six microarray datasets we sought to analyze (Figs. S1A**-**D and S2A-D). Next, differences in gene expression in smokers vs. non-smokers and asthmatics vs. healthy individuals were modeled using linear regression, including proband age, sex, sample type and dataset as covariates in the model. When modeling the age effect with linear regression, the age of probands for whom age information was not available was set to the average age of all other probands (separately for smokers’ datasets GSE63127 and asthmatics’ dataset GSE4302; for age distribution, see Figs. S1E and S2E). *P* values were next corrected for multiple hypothesis testing using the Benjamini-Hochberg correction. In post hoc analysis, log_2_ transformed data not corrected for age and sex were plotted with ggpubr [19], and two sided Mann-Whitney U tests were performed using the Wilcox.test function in R.

**Fig. 1:**
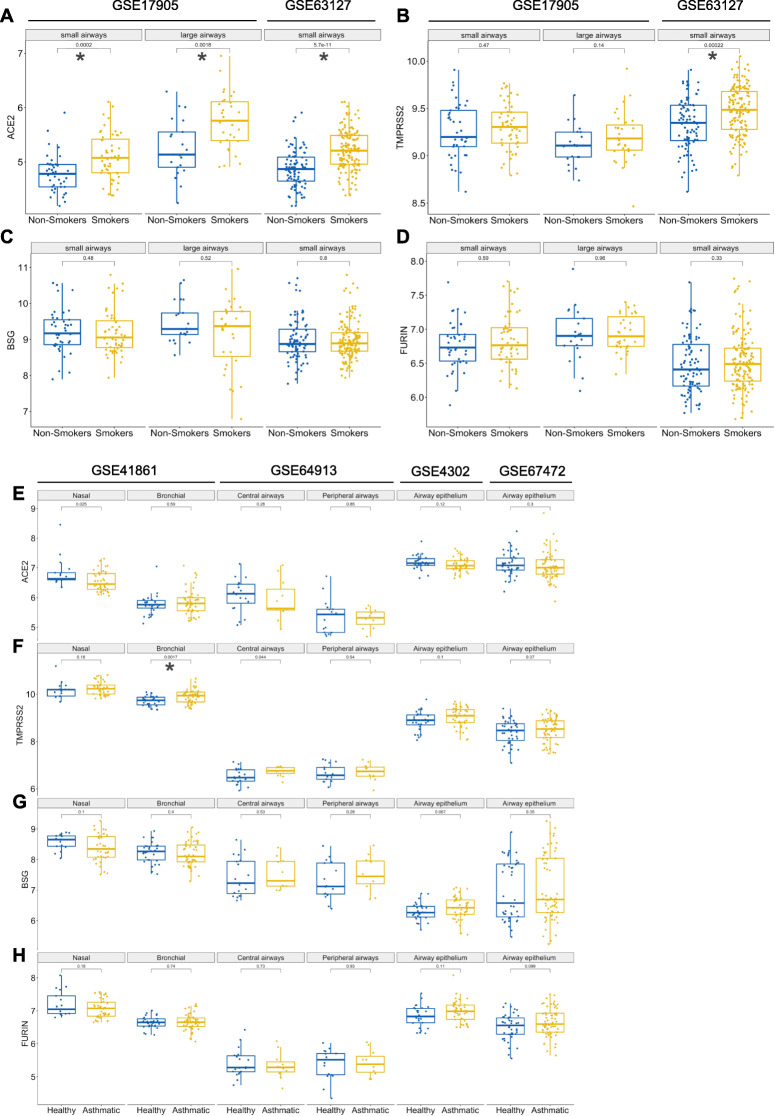
Expression of *ACE2*, *TMPRSS2*, *BSG* and *FURIN* in respiratory epithelium of smokers, asthmatics and healthy individuals. **a**-**d** Microarray datasets of bronchial brushings from healthy non-smokers and smokers. GSE17905: non-smokers small airways (*n* = 41), large airways (*n* = 21); smokers small airways (*n* = 52), large airways (*n* = 31). GSE63127: non-smokers (*n* = 87); smokers (*n* = 143). Gene expression for **a** *ACE2*, **b** *TMPRSS2*, **c** *BSG*, and **d** *FURIN*. **e**-**h** Microarray datasets of airway epithelial brushings from healthy controls (GSE41861, bronchial *n* = 30 and nasal *n* = 17; GSE64913, central airway *n* = 20 and peripheral airway *n* = 17; GSE4302, *n* = 28; GSE67472, *n* = 43) and asthma patients (GSE41861, bronchial *n* = 51 and nasal *n* = 40; GSE64913, central airway *n* = 11 and peripheral airway *n* = 11; GSE4302, *n* = 42; GSE67472, *n* = 62). Gene expression for **e** *ACE2*, **f** *TMPRSS2*, **g** *BSG*, and **h** *FURIN*. Data are shown as log_2_ transformed expression values not corrected for proband age and sex. Multiple comparison significance levels: *p < 0.002

Older age is an important risk factor for adverse COVID-19 outcomes [20]. Another risk factor is male sex [21]. Since there is a very high smoking rate in Chinese males compared to females—66.1% vs. 3.2% according to Ma et al. [22]—it has been suggested that smoking could be a risk factor for the more severe COVID-19 disease course observed in males [20, 23]; however, this topic is controversially discussed [24–26]. In our analysis, we therefore removed possible confounding effects of age and sex on the status of receptor mRNA expression by regressing the linear effects of age and sex and testing our hypothesis on model residuals (Table 1). For samples for which sex information was not available, sex was predicted based on the expression of both X inactive specific transcript (XIST; high expression in females) and ribosomal protein S4 Y-linked 1 (RPS4Y1; high expression in males) simultaneously. In post hoc analysis, we also plotted log_2_ transformed expression values not corrected for age and sex (Fig. 1). Consistent with previous reports [7, 27], we found significantly higher *ACE2* expression in airway epithelia from healthy smokers vs. healthy non-smokers (Fig. 1a**,** Table 1). Similarly, we found significantly higher *TMPRSS2* expression in smokers in one out of two datasets analyzed (Fig. 1b**,** Table 1), whereas *BSG* and *FURIN* expression did not significantly differ between smokers and non-smokers (Fig. 1c-d**,** Table 1). Taken together, these results indicate that, independently of sex and age, *ACE2* and *TMPRSS2* are upregulated in the airway epithelia of smokers. In addition, our analyses establish that *BSG* and *FURIN*, two alternate potential SARS-CoV-2 receptors, are expressed in the human respiratory tract.

**Table 1:** Modeling differences in *ACE2*, *TMPRSS2*, *BSG* and *FURIN* expression accounting for proband age and sex

gene	Adjusted ***p***-value smokers vs. non-smokers	*β* smokers vs. non-smokers	Adjusted p-value asthmatics vs. healthy	*β* asthmatics vs. healthy
*ACE2*	**6.80E-16**	0.36	0.186	−0.074
*TMPRSS2*	**0.00044**	0.1	**0.0156**	0.13
*BSG*	0.31	−0.065	0.59	0.038
*FURIN*	0.31	0.047	0.29	0.048

Adjusted *p*-values and linear regression model coefficients (*β*), testing hypotheses whether expression of *ACE2*, *TMPRSS2*, *BSG* and *FURIN* differ between smokers vs. non-smokers (columns 1,2) and asthmatics vs. healthy individuals (columns 3,4). The linear regression models were controlled for the linear effect of age, sex, sample type, and dataset

We next examined four RNA microarray datasets for *ACE2*, *TMPRSS2*, *BSG* and *FURIN* expression in airway epithelia from patients with a common respiratory disease, asthma. Patients with chronic respiratory disorders including asthma are considered a COVID-19 high-risk category [3]. Interestingly, we found that *ACE2* expression tended to be downregulated in nasal epithelium, whereas *TMPRSS2* was significantly upregulated in bronchi and central airways of asthmatics (Fig. 1e-f**,** Table 1). *ACE2* was proposed to be an interferon-stimulated gene [28]; therefore, a potential explanation for *ACE2* downregulation in asthmatics could be corticosteroid use. However, more recent data indicate that a novel, primate-specific *ACE2* isoform exists that is incapable of binding SARS-CoV-2, and that this isoform is interferon-stimulated, whereas the canonical *ACE2* is not [29, 30]. Further research is needed to address these highly interesting developments in more detail.

Additionally, we did not find any difference in *BSG* or *FURIN* expression between healthy and asthmatic individuals (Fig. 1g-h**,** Table 1). These findings point towards a possible differential regulation of *ACE2* and *TMPRSS2* expression in airway epithelia and warrant further investigation into the underlying mechanism.

**Fig. 2:**
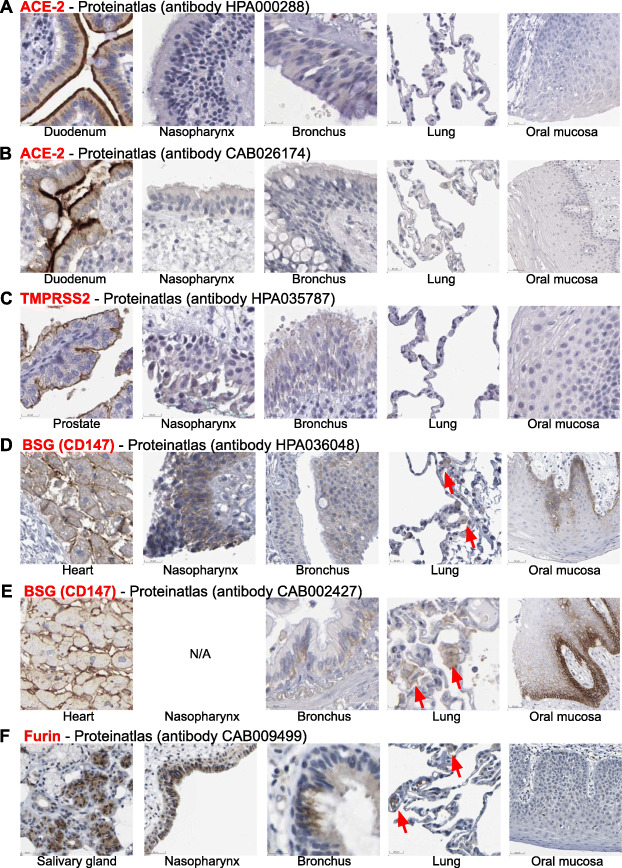
Expression of SARS-CoV-2 receptor and cofactor proteins in respiratory epithelium as analyzed by immunohistochemistry. Representative screenshots of immunohistochemistry (IHC) images from The Human Protein Atlas (www.proteinatlas.org). **a**-**b** ACE-2 IHC images from the nasopharynx (*n* = 4), bronchi (*n* = 3), lungs (*n* = 6), and oral mucosa (*n* = 4). Positive control: duodenum tissue (*n* = 6). **c** TMPRSS2 IHC images from the nasopharynx (*n* = 3), bronchi (*n* = 2), lungs (*n* = 3), and oral mucosa (*n* = 3). Positive control: prostate tissue (*n* = 3). **d**-**e** BSG/CD147 IHC images from the nasopharynx (*n* = 2), bronchi (*n* = 3), lungs (*n* = 6), and oral mucosa (*n* = 5). Positive control: heart tissue (*n* = 3). **f** Furin IHC images from the nasopharynx (*n* = 3), bronchi (*n* = 3), lungs (*n* = 3), and oral mucosa (*n* = 3). Positive control: salivary gland tissue (*n* = 3). Red arrows indicate IHC positive cells morphologically consistent with alveolar macrophages. Scale bars, lower left of respective images. Images were analyzed by a board-certified surgical pathologist (C.M.S.)

We next aimed to compare RNA expression of these receptors and cofactors to protein expression. Therefore, we examined immunohistochemistry (IHC) images from respiratory and other tissues on The Human Protein Atlas [31]. ACE-2 IHC staining was strong in epithelial cells of the duodenum and was found in other organs including testis and kidney. In contrast, human respiratory epithelial cells in samples from the nasopharynx, bronchi and lungs, as well as squamous epithelial cells from the oral mucosa, were completely negative for ACE-2 staining by IHC with two different antibodies (Fig. 2a-b). Similar negative IHC staining results were also observed for TMPRSS2 protein (Fig. 2c). In contrast, basigin protein was widely expressed in human tissues including heart muscle, brain, liver and kidney, and, importantly, was positive in respiratory epithelial cells from the nasopharynx and bronchi (Fig. 2d-e). Similar to ACE-2 and TMPRSS2, basigin was negative in alveolar epithelial cells but showed multifocal positivity in cells morphologically consistent with alveolar macrophages (Fig. 2d-e). Furthermore, basigin was strongly expressed in the oral mucosa, with a gradient from basal to apical epithelial cells (Fig. 2d-e). Finally, furin IHC stainings were positive in nasopharynx and bronchial epithelial cells, and negative in alveolar epithelial cells, with focal positivity in alveolar macrophages (Fig. 2f). In summary, basigin and furin protein were expressed in airway epithelia of nasopharynx and bronchi, whereas ACE-2 and TMPRSS2 protein stainings were negative.

Our findings are in line with and extend recent studies addressing SARS-CoV-2 receptor and cofactor expression in the respiratory tract [32, 33], but are in stark contrast to a 2004 study by Hamming et al. [34], who found strong and widespread ACE-2 expression in alveolar epithelial cells and basal epithelial cells of the nasopharynx and oral mucosa. Our study highlights the discrepancies between RNA and protein expression of these receptors and cofactors, and points towards potential issues with IHC staining reproducibility and antibody specificity, important factors that need to be addressed in future investigations. One limitation of our study was that in The Human Protein Atlas, only small numbers of IHC stained samples for each tissue and molecule analyzed were available. Therefore, further studies exploring the protein expression and cellular localization of SARS-CoV-2 receptors and cofactors in each of these tissue types, ideally using tissue microarrays with large numbers of samples from multiple donor cohorts, and using multiple different antibodies, are warranted. In addition, these studies should use the recently developed multiplexed microscopy technologies [35] to address protein co-expression patterns and better delineate the cellular subsets expressing these SARS-CoV-2 entry receptors and cofactors.

## Supplementary information

**Additional file 1.**

## Data Availability

All data presented are from publicly available datasets, as detailed in the manuscript and supplemental information. Datasets for smokers vs. non-smokers: https://www.ncbi.nlm.nih.gov/geo/query/acc.cgi?acc=GSE17905 https://www.ncbi.nlm.nih.gov/geo/query/acc.cgi?acc=GSE63127 Datasets for asthmatics vs. healthy individuals: https://www.ncbi.nlm.nih.gov/geo/query/acc.cgi?acc=GSE41861 https://www.ncbi.nlm.nih.gov/geo/query/acc.cgi?acc=GSE64913 https://www.ncbi.nlm.nih.gov/geo/query/acc.cgi?acc=GSE4302 https://www.ncbi.nlm.nih.gov/geo/query/acc.cgi?acc=GSE67472 Datasets for receptor protein immunohistochemistry: https://www.proteinatlas.org/ENSG00000130234-ACE2/tissue https://www.proteinatlas.org/ENSG00000184012-TMPRSS2/tissue https://www.proteinatlas.org/ENSG00000172270-BSG/tissue https://www.proteinatlas.org/ENSG00000140564-FURIN/tissue

## Abbreviations

SARS-CoV-2: Severe acute respiratory syndrome coronavirus 2
COVID-19: Coronavirus disease 2019
ACE-2: Angiotensin I converting enzyme 2
TMPRSS2: Transmembrane serine protease 2
BSG: Basigin
XIST: X inactive specific transcript
RPS4Y1: Ribosomal protein S4 Y-linked 1
