# Supplementary material for: Expression of SARS-CoV-2 entry receptors in the respiratory tract of healthy individuals, smokers and asthmatics
Source: Respir Res. 2020 Sep 29;21:252. doi: 10.1186/s12931-020-01521-x (PMC7523260; doi:10.1186/s12931-020-01521-x)
Supplement: Supplementary file 1 — Additional file 1. [file 12931_2020_1521_MOESM1_ESM.pdf]

Supplemental Information

Detailed description of the samples from microarray datasets used

Smokers datasets:

1. GSE17905 contains samples of bronchial brushings from non-smokers: small airways (n=41), large airways (n=21); and smokers: small airways (n=52), large airways (n=31).
2. GSE63127 contains samples from non-smokers (n=87) and smokers (n=143).

There are 12 overlapping samples between GSE17905 and GSE63127 datasets. These 12 overlapping samples were removed from the GSE17905 dataset in our analysis.

Asthma datasets:

1. GSE41861 contains epithelial brushings from bronchi and nose from healthy controls: bronchial n=30 and nasal n=17; and asthma patients: bronchial n=51 and nasal n=40.
2. GSE4302 contains airway epithelial brushings from n=28 healthy controls and n=42 patients with asthma. This dataset includes airway samples from 42 subjects with asthma of which a subgroup of 32 subjects completed a randomized placebo-controlled trial of fluticasone propionate. We only analyzed samples of subjects with asthma at baseline before they entered the trial (n=42) and compared them to samples of healthy individuals.
3. GSE64913 contains samples of airway brushings from healthy controls: central airway n=20 and peripheral airway n=17; and asthmatic patients: central airway n=11 and peripheral airway n=11.
   There are more samples listed in the GEO GSE64913 repository than are analysed in our manuscript. The reason for not including 5 normal and 6 asthmatic samples which are listed in the GEO repository is that the raw CEL data for those samples are missing from GEO repository. We only downloaded and reanalysed CEL files that were available in the GEO website:
   https://www.ncbi.nlm.nih.gov/geo/download/?acc=GSE64913&format=file

   A list of CEL files that are missing from the GEO GSE64913 repository:
   GSM1583182 (Healthy_Central_Patient_1),
   GSM1583190 (Healthy_Central_Patient_9),
   GSM1583199 (Healthy_Central_Patient_18),
   GSM1583213 (Healthy_Peripheral_Patient_13),
   GSM1583217 (Healthy_Peripheral_Patient_17),
   GSM1583228 (Severe_Central_Patient_29),
   GSM1583230 (Severe_Central_Patient_34),
   GSM1583241 (Severe_Peripheral_Patient_29),
   GSM1583243 (Severe_Peripheral_Patient_31),
   GSM1583246 (Severe_Peripheral_Patient_35),
   GSM1583248 (Severe_Peripheral_Patient_37)

4. GSE67472 contains samples of airway brushings from healthy controls n= 43 and asthmatic patients n= 62.

Supplemental Figures and Legends

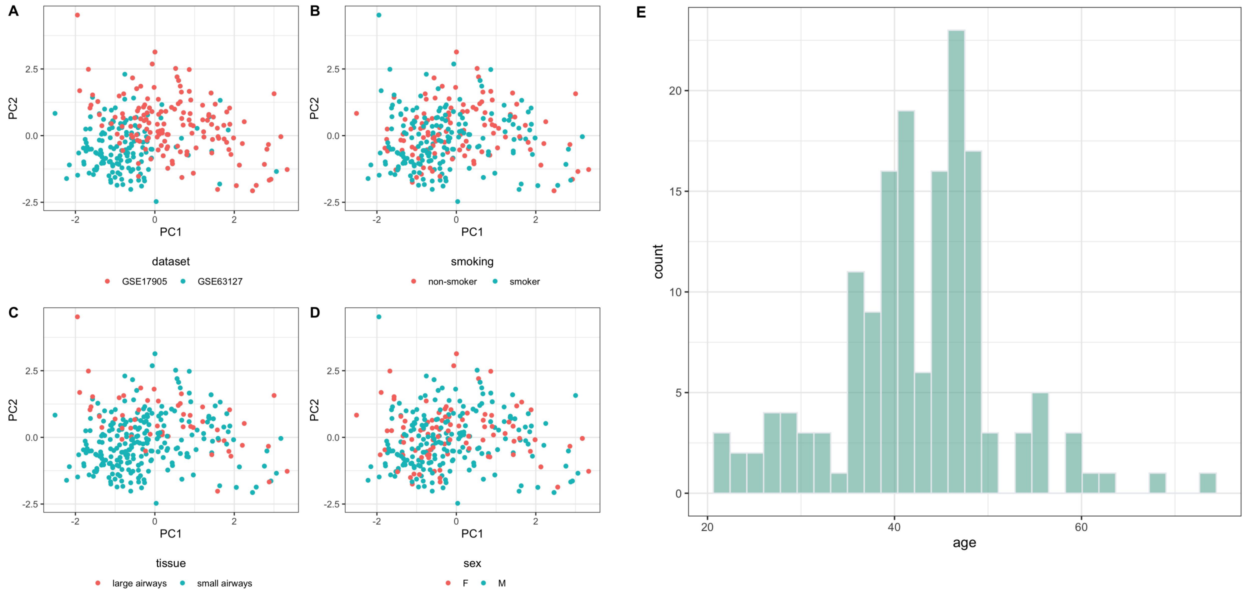

Figure S1. PCA of gene expression and age distribution of healthy smokers and non-smokers.

(A-D) PCA of microarray datasets of bronchial brushings from healthy smokers and non-smokers. GSE17905: non-smokers small airways (n=41), large airways (n=21); smokers small airways (n=52), large airways (n=31). GSE63127: non-smokers (n=87); smokers (n=143). PCA was computed on expression of *ACE2*, *TMPRSS2*, *BSG* and *FURIN* and colored by **(A)** datasets, **(B)** smoking status, **(C)** tissue type and **(D)** sex. **(E)** The age distribution of smokers and healthy non-smokers from GSE17905 and GSE63127 datasets. Individuals in dataset GSE63127 for whom age information was not available were omitted.

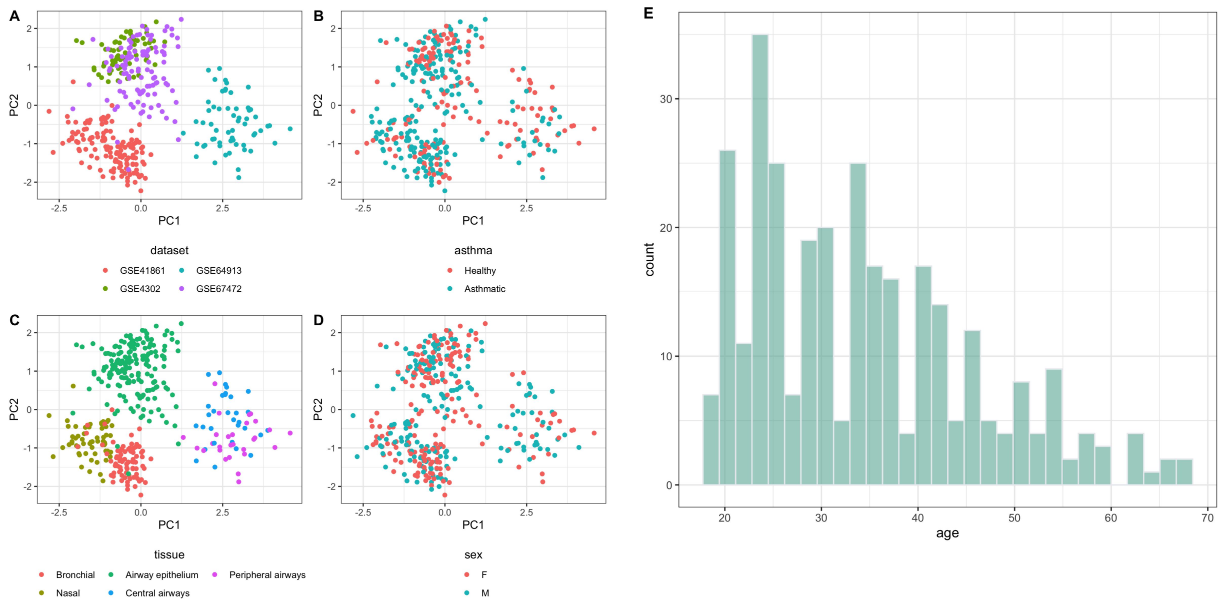

Figure S2. PCA of gene expression and age distribution of asthmatics and healthy individuals.

(A-D) PCA of microarray datasets of airway epithelial brushings from healthy controls (GSE41861, bronchial n=30 and nasal n=17; GSE64913, central airway n=20 and peripheral airway n=17 ; GSE4302, n=28; GSE67472, n=43) and asthma patients (GSE41861, bronchial n=51 and nasal n=40; GSE64913, central airway n=11 and peripheral airway n=11; GSE4302, n=42; GSE67472, n=62). PCA was computed on expression of *ACE2*, *TMPRSS2*, *BSG* and *FURIN* and colored by **(A)** datasets, **(B)** asthma vs. healthy, **(C)** tissue type and **(D)** sex. **(E)** The age distribution of asthmatics and healthy individuals from GSE41861, GSE64913 and GSE67472 datasets. Age information was not available for GSE4302 and was omitted.
